# Individualized, low-cost and accessible pulmonary rehabilitation program based on functional clinical tests for individuals with COPD—a study protocol of a randomized controlled trial

**DOI:** 10.1186/s13063-021-05267-9

**Published:** 2021-05-26

**Authors:** Marcela Maria Carvalho da Silva, Juliano Ferreira Arcuri, Valéria Amorim Pires Di Lorenzo

**Affiliations:** Postgraduate Physiotherapy Department of Federal University of São Carlos, Rodovia Washington Luiz, São Carlos, São Paulo 13565-905 Brazil

**Keywords:** Rehabilitation, Exercise therapy, COPD, Physical functional performance, Randomized controlled trial

## Abstract

**Background:**

Patients with chronic obstructive pulmonary disease (COPD) present pulmonary and extrapulmonary impairments. The strategies used to mitigate these impairments are pulmonary rehabilitation programs (PRP). However, there is limited access to PRP in specialized centers and the study of low-cost home rehabilitation programs had non-individualized prescription, which might have led to insignificant positive effects. So, it is important to develop new low-cost protocols that involve individualized prescription, as well as physiotherapist supervision. This study describes an accessible, low-cost, and individualized pulmonary rehabilitation protocol and compare its results when performed with or without a weekly physiotherapist-supervised session on patients with COPD.

**Methods:**

This is a descriptive protocol of a clinical trial, randomized, single-blinded, and type of framework is superiority conducted at the Spirometry and Respiratory Physical Therapy Laboratory of the Federal University of São Carlos (UFSCar). The trial is registered in the Brazilian Clinical Trials Registry (ReBec) URL: http://www.ensaiosclinicos.gov.br/rg/RBR-533hht/ with Register Number UTN code U1111–1220-8245. The sample size is 50 patients and is calculated using the results of a pilot study.

**Discussion-potential impact and significance of the study:**

It is expected that the low-cost and new supervised rehabilitation program complemented with home exercises will present positive results, especially on exercise capacity, which will make available a more accessible and effective PRP for patients with COPD.

**Trial registration:**

ClinicalTrials.gov U1111-1220-8245. Registered on September 20, 2018.

**Supplementary Information:**

The online version contains supplementary material available at 10.1186/s13063-021-05267-9.

## Administrative information

**Table:** 

Title {1}	Individualized, low-cost, and accessible pulmonary rehabilitation program based on functional clinical tests for individuals with COPD—a study protocol of a randomized controlled trial
Trial registration {2a and 2b}.	UTN code U1111-1220-8245
Protocol version {3}	This is the first and definitive protocol version. Participants will be recruited between September 20, 2018, and prevision is June 2021. Study completion is expected to be August 2021. The study protocol has been submitted before the end of the recruitment and before the last patient.
Funding {4}	Regular Project funding by the São Paulo State Research Foundation (FAPESP). FAPESP Process: 2018/06970-5 (Additional file 5) and Coordination for the Improvement of Higher Education Personnel (CAPES). The study is funded by FAPESP, and it will not have any role in the study design, collection, management, data analysis, and interpretation. Moreover, it will not have authority to change any aspect of the protocol or in writing the manuscript.
Author details {5a}	*All authors: Postgraduate Physiotherapy Department of Federal University of São Carlos, Brazil*
Name and contact information for the trial sponsor {5b}	Valéria Amorim Pires Di Lorenzo is a coordinator Contact: vallorenzo@ufscar.br
Role of sponsor {5c}	Valéria Amorim Pires Di Lorenzo is responsibility to initiate and manage the clinical.

## Introduction

### Background, rationale {6a}

Patients with chronic obstructive pulmonary disease (COPD) present pulmonary impairments, such as dyspnea, pulmonary hyperinflation, gas exchange deficit, and decreased respiratory-muscle endurance and strength. Moreover, extra pulmonary manifestations on the skeletal muscles, cognitive, nutritional, and cardiovascular functions lead to reduced physical-activity levels, exercise capacity, functionality and quality of life, and ultimately increased morbidity and mortality [1–3].

In order to mitigate these impairments, pulmonary rehabilitation programs (PRP) aim to increase autonomy for physical, social and emotional performance, positively influencing symptoms, disease progression, and increasing exercise tolerance. Hence, quality of life, exercise capacity, and psychological condition are improved and morbidity, health costs, and mortality are reduced [4, 5].

In spite of the benefits attributed to PRP, less than 10% of patients diagnosed with COPD have access to a specialized service, and there are several reasons related to this, such as the lack of rehabilitation centers specially in the countryside and rural areas, displacement difficulties due to poor physical conditions and mobility limitations and family dependency. Furthermore, the high cost in construction and maintenance of these centers [6] decreases accessibility and feasibility. Therefore, alternative strategies have been investigated to conduct a PRP.

Different technological levels are some of the possible strategies to address this matter. High technological assistance, such as telerehabilitation, are characterized by home-based activities performed by patients with professional supervision using communication technologies like computers, Internet, and remote control and monitoring. Thus, no patient dislocation is needed; nevertheless, the high cost prevents its feasibility to low-income populations [7].

Low-level technologies (with low cost) are also described in the literature and they include educational charts, domiciliary exercise instructions and functional physical activities [8]. The advantage of this method is the feasibility, since they can be conducted in primary care, low-technology rehabilitation centers or at the patient’s home. Previous studies have verified the effect of low-cost pulmonary rehabilitation performed in the patients’ home and supervised by a physical therapist, and they concluded that domiciliary PRPs are appropriate alternatives [6, 9, 10]. Nevertheless, in most studies, exercise prescription is not individualized, and additional results could be found, since the empirical protocol may underestimate patient’s capacity, or overestimate, leading to muscle soreness, fatigue, and the patients possibly abandoning the treatment. Hence, the lack of individualized prescription is not compatible with the marked heterogeneity of intervention and disease phenotypes [6, 11].

Furthermore, although previous studies [6, 10] did not find differences between a low-cost home and center-based rehabilitation, both groups did not achieve minimal clinical difference in the Borg scale [6], and distance walked on the 6-min walk test (6MWT). Moreover, the increase in exercise prescription is not the same in both rehabilitation groups, which leads to the lack of interpretation of results, which are influenced not only by the environment where it is executed but also by the exercise prescription in the PRP.

Therefore, considering the limited access to PRP in specialized center and that the study of low-cost home rehabilitation programs had non-individualized prescription, which may have led to insignificant positive effects, it is important to develop new low-cost protocols that include individualized prescription, and the effect of physiotherapist supervision.

### Objectives {7}

So, the objective of the present study is to describe an accessible, low-cost, and individualized pulmonary rehabilitation protocol and compare its results when performed with or without a weekly physiotherapist-supervised session on patients with COPD.

### Trial design {8}

This is a descriptive protocol of a clinical trial, randomized, single-blinded, and type of framework is superiority.

## Methods: participants, interventions, and outcomes

### Study setting {9}

**Fig. 1:**
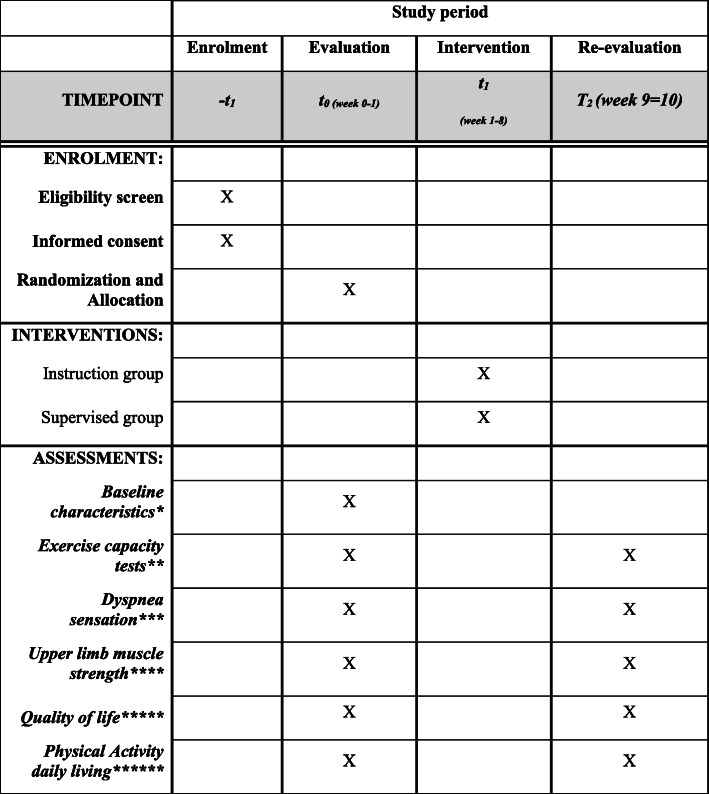
Standard Protocol Items: Recommendations for Interventional Trials (SPIRIT) checklist providing information about volunteer recruitment and variables evaluated in each period according to SPIRIT recommendations. *Baseline characteristics include pulmonary function assessed by spirometry, information about the patients’ anthropometric data, educational level, presence of continuous use of oxygen therapy, ongoing pulmonary rehabilitation, active smoking, and comorbidities (Charlson Comorbidity Index). ***Exercise capacity test includes 6-min step test, 6-min walk test, and 1-min sit-to-stand test. *** Dyspnea sensation includes Modified Medical Research Council (mMRC). ****Upper limb muscle strength includes ten repetition maximum test. *****Quality of life includes The Saint George Respiratory Questionnaire (SQRQ). ******Physical activity daily living includes actigraph

The study protocol is developed according to Standard Protocol Items: Recommendations for Interventional Trials (SPIRIT) 2013 Checklist guidelines (Additional file 1 and Fig. 1), and the trial is registered at URL: http://www.ensaiosclinicos.gov.br/rg/RBR-533hht/ with Register Number RBR-533hht or UTN code U1111-1220-8245. Thus, the trials status is the registration date and initial data is on September 20, 2018, and the actual status is ongoing and the prevision is in June 2021.

### Eligibility criteria {10}

The inclusion criteria are (i) age between 50 and 85 years, (ii) diagnosis of COPD, (iii) absence of exacerbation and stability of drug treatment in the last 4 weeks, (iv) absence of PRP in the last 6 months, (v) Modified Medical Research Council (mMRC) greater than or equal to two, and (vi) low exercise capacity verified by the results of 6-min step test and/or 6-min walk test (lower than 80% of predicted value)—lack of cognitive impairment with language deficiency that impairs understanding the verbal instructions.

The exclusion criteria are conditions that impair executing the proposed tests [12], such as unstable angina and myocardial infarction in previous months, arrhythmias, unstable blood pressure, and body-mass index greater than 35 kg/m^2^; malignant cancer treatment; orthopedic, rheumatic, neuromuscular, and visual problems that make it impossible to walk and/or go up and down stairs.

### Who will take informed consent? {26a}

On the consent form, at the first contact (first day of evaluation), the participants will be asked by the main researcher if they agree to participate of the study. The main researcher is responsible by recruiting the patient and gaining informed consent. The main researcher is not involved by randomization, evaluation, or intervention.

### Additional consent provisions for collection and use of participant data and biological specimens {26b}

Participants will also be asked for permission by researcher team to share relevant data with people from the universities taking part in the research or from regulatory authorities, where relevant. This trial does not involve collecting biological specimens for storage.

## Interventions

### Explanation for the choice of comparators {6b}

The comparators are choice because they are low-cost instruments to assess the effects of intervention and they are validated for COPD.

### Intervention description {11a}

The intervention will last 8 weeks and will start 1 week after the assessment has been concluded. The intervention has two distinct moments: personalized instructions and exercise prescription. The exercise prescription is based on the performance in functional physical tests and it consists of warm up, aerobic exercise, muscle strengthening, and stretching. The exercise load is progressive according to individual tolerance and performance in clinical tests, and according to the protocol created by our researcher group, based on previous studies [4, 6].

#### Personalized instructions (booklet)

On the first meeting with the physiotherapist responsible for the rehabilitation, all patients in both groups will participate in one educational session organized in a booklet that contains instructions regarding COPD physiopathology, pulmonary and extrapulmonary manifestations, multidisciplinary treatment, involving smoking cessation, correct medication administration, vaccination, symptom control, and benefits of practicing physical activity (Additional file 2). Furthermore, patients will be instructed about the signals to interrupt exercise, exercise safety, and symptom control (Additional file 2) and environmental changes to facilitate exercise practices.

#### Warm up

This phase is composed of mild intensity physical activity (Ex. Light walk) for 3 to 5 min (see progression in Additional file 3).

#### Aerobic exercise

This phase includes more vigorous walking, sit-to-stand movements, and step up and down. The weekly progression of these exercises (Additional file 3) will consider the following variables for all patients: (i) the results from maximum tolerance in the assessed functional physical tests (6MWT, 6MST, and 1MSST): Initially, a suggested exercise intensity/volume of 80% of the verified performance in the assessed functional physical tests will guide exercise training, the weekly progression will achieve up to 100% of that performance (see progression in Additional file 3); (ii) symptom-limited: during the execution of the exercise at the suggested intensity/volume, patients shall maintain Borg scale in a range of 3 to 5 points (moderate to intense effort perception), they will be instructed to increase intensity/volume if Borg is under that range or decrease it if Borg is above that range; and (iii) Duration: the duration of each exercise will be increased every week, so the patient will increase walking time from 5 to 20 min, stepping up and down from 3 to 15 min and sitting and standing from 1 to 3 min (see progression in Additional file 3). If a patient does not have a step (20–25 cm), the researchers will provide one during the research period. Additionally, patients will be instructed to walk on a flat floor (backyard, hallway, or street).

Upper limbs strengthening: The load for upper limbs strengthening will vary from 50 to 100% of the verified load in the 10-maximum repetition test performed in the diagonal movement (see progression in Additional file 3). To execute the exercises at home, patients will be instructed to use food packages and the load will be progressively increased every week. There will also be a variation in the number of sets (from two to four) and repetitions (from 10 to 15) (see progression in Additional file 3). If they do not have the food packages to perform the exercise at home, the researchers will provide free weights.

#### Stretching

Stretching will be performed on the cervical region, upper and lower limbs, according to the model illustrated in the educational booklet (Additional file 2). Patients will be instructed to remain in each position for 20 s.

#### Relaxation

Patients will be seated in a relaxed position for 5 to 10 min with relaxation music and calm breathing. The information on the intensity, progression, and duration of each prescribed step, as well as the activity performed at home, will be recorded in the individual monitoring form (Additional file 3).

### Action for randomized groups

#### Instruction group (IG)

Patients will have one physiotherapist-supervised session, which will be the first day of PRP. In that session, this group will receive a personalized booklet that will be read together with the physiotherapist and all exercises will be explained and performed together with the physiotherapist and they will be oriented self monitoring of vital signs and symptoms. On the next 7 weeks of PRP, the patient will execute the exercises at home with equipment they have at home (chair, step, food packages) and if they have any questions/doubt, they to call the physiotherapist. In addition, patients will record exercise habits on a diary during the week and they will be asked to return the information at the end of the 8-week period.

#### Supervised group (SG)

Patients will receive the same booklet as the IG (education session) and, for an 8-week period, they will have a weekly supervised physiotherapy session (at laboratory) where they will perform the same training they perform at home. All patients will be encouraged to exercise at home following the booklet four times every week and they will be oriented self monitoring of vital signs and symptoms .

During the supervised sessions, patients will be monitored regarding pulse oximetry and Borg scale, recording the values on a vital sign monitoring form (Additional file 4) and the clinical evolution form will be filled out by a physiotherapist.

At the end of PRP, patients of both groups will undergo reassessment (the same executed pre-rehabilitation, except for spirometry).

### Criteria for discontinuing or modifying allocated interventions {11b}

Patients will be instructed to receive general exercise instructions to maintain a regular medication schedule and refrain from exercising after lunch or dinner, or if they perceive symptoms of infection, fever, dizziness, or thoracic pain. If the patients experience discomfort, they should seek a health professional.

### Strategies to improve adherence to interventions {11c}

The patients will be instructed to record exercise habits on a diary during the week, and they will be asked to return the information at the end of the 8-week period; furthermore, the educational session will address the issue of adherence. Outcome data is to be collected for participants who discontinue from intervention protocols and the reason why the patient discontinued and how long the patient completed the protocol. The presence of 75% in the session will be considered a positive adherence.

### Relevant concomitant care permitted or prohibited during the trial {11d}

Implementing accessible, low-cost and individualized pulmonary rehabilitation with or without a weekly physiotherapist-supervised session for patients with COPD will not require alteration to usual care pathways (including use of any medication) and these will continue for both trial arms. It is important to emphasize that all patients will be oriented to continue on the drug regime.

### Provisions for post-trial care {30}

There is no anticipated harm and compensation for trial participation.

### Outcomes {12}

**Fig. 2:**
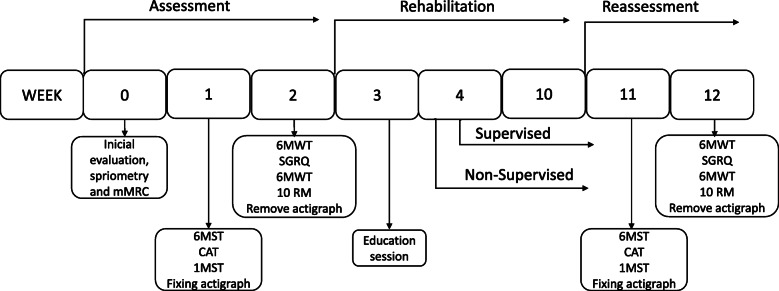
Experimental design of study. mMRC Modified Medical Research Council, 6MWT 6-min walk test, IMST 1-min sit-to-stand test, 6MST 6-min step test, 10RM Ten repetition maximum test, SGRQ Saint George Respiratory Questionnaire, CAT COPD Assessment Test

The patient’s assessment will be conducted in a standard sequence, presented in Fig. 1 (Participant timeline) and Fig. 2 and the assessment will be conducted in two visits, with at least 7 days between them. The procedure will also be executed before and after PRP to verify the effects of the program.

The baseline characteristics of this study will be information on the patients’ anthropometric data, educational level, continuous use of oxygen therapy, ongoing pulmonary rehabilitation, active smoking, comorbidities (Charlson Comorbidity Index), and pulmonary function assessed by spirometry [13].

The primary outcome of this study will be the number of steps in the 6-min step test (6MST) (will be executed on first day of evaluation), which will also be used to guide aerobic training prescription. A single 6MST trial will be performed using the standardized technique [14, 15], and the number of steps will be registered and compared with normative values [16].

The secondary outcomes will be:

(i) *Six-minute walk test:* Two 6MWTs (will be executed on second day of evaluation) will be performed using the standardized technique [12]; for statistical analysis, we will consider the greatest distance walked between the two tests. The distance will be compared with normative values [17].
(ii) 1-*min sit-to-stand test:* 1MST (will be executed on first day of evaluation) will be performed using the standardized technique [18] and the number of sit-to-stand will recorded and compared with normative values [19]
(iii) *Modified Medical Research Council (mMRC):* Is as a questionnaire graded from 0 to 4 points and the higher the score the worst the dyspnea [20] (will be executed on first day of evaluation).
(iv) *Ten repetition maximum test:* will be performed with diagonal movements of dominant upper limb with incremental free weight [21] (will be executed on second day of evaluation)
(v) *Quality of life*: The Saint George Respiratory Questionnaire (SQRQ) (will be executed on second day of evaluation) and COPD Assessment Test (CAT) (will be executed on first day of evaluation) [22] will be executed by the same evaluator using an interview format.
(vi) *Physical Activity daily living (PADL)*: ActiGraph activPAL3TM (PAL Technologies Ltd., Glasgow, Reino Unido) will be affixed (Tegaderm-3 M) in the middle of the right thigh [23] for 7 days (between the first and the second days of evaluation). The main variables will be average daily sitting time and standing time (7 days), daily number of steps, and exercise intensity [24]

### Participant timeline {13}

#### Sample size {14}

The sample size is calculated using the results of a ten-patient pilot study, and considering an effect size of 0.33, an expected power of 80% and alpha-error 5%, 36 patients will be needed and dropout is considered 20%, a total of 50 patients. For the missing data, multiple imputations followed by a sensitivity analysis will be conducted. The pilot study is unpublished in other ways of publication.

#### Recruitment {15}

Patients undergoing medical treatment at public outpatient pulmonology services in São Carlos will be invited to participate in the research according to eligibility criteria. After acceptance, all participants will sign a consent form and will undergo a spirometry test [13] (on the first day of evaluation), and clinical and physical evaluation (on the second and the third day of evaluation) at the Spirometry and Respiratory Physiotherapy laboratory of UFSCar.

#### Assignment of interventions: allocation

### Sequence generation {16a}

The allocation sequence is generated by an advisor who is neither the intervention physiotherapist nor the evaluator, using an online program (Random.org) that generates the sequence of numbers for randomization patients that will be randomized in two groups: instruction group (IG) and supervised group (SG).

### Concealment mechanism {16b}

The results of sequence will be stored in a sealed envelope and kept confidential by this advisor, who will consult by the intervention physiotherapist by phone.

### Implementation {16c}

The advisor will consult the randomization. The intervening physiotherapist was only aware of the allocation at the end of the pre-intervention evaluations. The evaluating physiotherapist did not have access to the patient’s allocation throughout the study.

### Assignment of interventions: blinding

#### Who will be blinded {17a}

The sequence of randomization will be generated and kept by the researcher not be involved in the recruitment, evaluation, or training of patients. The assessor will be blind to group allocation.

The protocol will be executed by a single training physiotherapist who is not involved in the evaluation or randomization process.

#### Procedure for unblinding if needed {17b}

Once patients are not blinded to the treatment, as well as the physiotherapists responsible for the treatment, reasons to unblind the assessors are not predicted in the present protocol.

### Data collection and management

#### Plans for assessment and collection of outcomes {18a}

The primary and secondary outcomes are described at “outcomes,” and the data collection forms can be found with the corresponding author.

#### Plans to promote participant retention and complete follow-up {18b}

The educational session will address the issue of adherence, and the patients will be instructed to record home exercise habits.

#### Data management {19}

Data entry will be managed by assessor blinded (not involved by rehabilitation), and the data will be registered in a documents clouds.

#### Confidentiality {27}

All personal information about potential and enrolled participants will be confidential. This information will be not shared; however, it will be consulted by researchers of this study. Finally, the information will be used for scientific purposes, always safeguarding their privacy before, during, and after the trial.

### Plans for collection, laboratory evaluation, and storage of biological specimens for genetic or molecular analysis in this trial/future use {33}

This trial does not involve collecting biological specimens for storage.

## Statistical methods

### Statistical methods for primary and secondary outcomes {20a}

Data entry will be managed by a blinded assessor (not involved in rehabilitation), and the data will be recorded in a documents clouds. All data will be analyzed by Statistical Package for the Social Sciences (SPSS) version 21.0. Two-way mixed effect ANOVA will be used to compare intergroup e intragroup (pre- and post-intervention) changes, considering significant a *p* value < 0.05.

### Interim analyses {21b}

The three main researchers will have access of the interim analyses, and they will have access to these interim results and make the final decision of the trial.

### Methods for additional analyses (e.g., subgroup analyses) {20b}

Two-way mixed effect ANOVA will be used to compare intergroup e intragroup (pre- and post-intervention) changes for additional analyses, considering significant a *p* value < 0.05.

### Methods in analysis to handle protocol non-adherence and any statistical methods to handle missing data {20c}

For the missing data, multiple data imputation will be used, performing 20 different random imputations and verifying the constancy of the differences found between the groups, a result will be considered consistent if most of the imputations led to a result according to the study hypothesis.

### Plans to give access to the full protocol, participant level-data, and statistical code {31c}

The datasets analyzed during the current study are available from the corresponding author on reasonable request.

## Oversight and monitoring

### Composition of the coordinating center and trial steering committee {5d}

The coordinating center is situated at the Spirometry and Respiratory Physical Therapy Laboratory of the Federal University of São Carlos (UFSCar) and comprises laboratory researchers. They are responsible for all local organization aspects such as identifying potential recruits, orientation support, and minimal equipment and room structure.

The Trial Steering Committee (TSC) consists of two laboratory researchers who are responsible for supervising the trial, as for instance concealment of blind allocation and evaluation of how the trial is conducted. They will meet once a month. There is no public Involvement Group (SPIG).

### Composition of the data monitoring committee, its role and reporting structure {21a}

Data monitoring committee is not considered as this is a low-risk intervention.

### Adverse event reporting and harms {22}

Adverse events (AEs) are not anticipated. The potential minor AEs that may be anticipated are fatigue, tachycardia, or blurred vision during the exercise that will be minimized by monitorization before and after the assessment and intervention. If the symptoms are persistent, the physiotherapist will immediately stop the exercise and conduce the first aid.

All protocol violations and all adverse-events could be registered and reported in the final paper and the adverse event could be report by the Ethics Committee of the Federal University of São Carlos.

### Frequency and plans for auditing trial conduct {23}

The trial Steering Committee, consisting of two laboratory researchers, will meet once a month to review how the trial is conducted.

Independent data monitoring is not considered as this is a low-risk intervention.

### Plans for communicating important protocol amendments to relevant parties (e.g., trial participants, ethical committees) {25}

The plans to communicate important protocol modifications will be registered for all relevant parties (REBEC (trial registries, Ethics Committee of the Federal University of São Carlos investigators, trial participants, journals, and financial support).

### Dissemination plans {31a}

The results will be communicated to scientific events, such as conferences, congresses, symposia, and social media. The data collected will be used for scientific purposes, always safeguarding their privacy before, during, and after the trial.

## Discussion

It is noteworthy that, as far as we know, this is the first study to show the effect of a low-cost, home-based physical rehabilitation associated with the educational and supervised program to improve physical capacity, dyspnea, and quality of life in patients with COPD. Previous studies [25, 26] have brought the importance and the concept in developing alternative rehabilitation programs; however, there are still no protocols that encompass accessibility (low cost), individualized, and weekly progressive prescription and which compare different supervision levels by the physiotherapist, in symptomatic patients with COPD.

### Thus, the potential impact and significance of the study: impact and significance of the study

It is expected that a low-cost once a week supervised rehabilitation program complemented with home-based exercises will present positive results, especially on exercise capacity, which will make available a more expanded and effective PRP for patients with COPD.

### Contribution and clinical applicability

The main contributions will be a more accessible alternative rehabilitation protocol for patients with COPD patients, which will also include individualized prescription; hence, more effective than previous protocols. Therefore, accessibility is a key point to expand rehabilitation opportunities so that more COPD patients can perform PRP in an alternative location, like at home, basic health unit, or community. We expected that the protocol could be adapted and applied in different situations, such as during the vacation period at University’s outpatient unit or in different populations, such as liver transplantation, chronic kidney disease, and others lung diseases.

### Other important information about the study

It is also expected that not only will we verify the pre- and post-hospitalization effect, but will also consider the minimum clinically important difference in all applied tests; thus, further confirming the effect of the intervention. In addition, in the post-intervention reevaluation of IG patients, they will be asked about adherence to weekly exercise, and this information will be recorded in medical records. SG patients will also be asked weekly about the frequency of the exercises performed.

Additional information worth noting is that all patients will receive medical support while participating in the study and can be referred to evaluation and conduction at any time.

### Strengths and limitations of the study

A limitation refers to the absence of lower limbs strengthening justified by the focus of the protocol that is based on functional physical tests.

## Trial status

This is the first and definitive protocol version. The trial is registered at URL: http://www.ensaiosclinicos.gov.br/rg/RBR-533hht/ with Register Number UTN code U1111-1220-8245. The registration date and initial data is September 20, 2018; the actual status is ongoing; and June 2021 is the estimated conclusion date. Study completion is expected to be August 2021. The protocol is approved by the Research Ethics Committee of the Federal University of São Carlos (UFSCar) number 4.348.948, CAAE: 85901318.0.0000.5504.

The study protocol has been submitted before the end of the recruitment and before the last patient.

## Supplementary Information

**Additional file 1.** SPIRIT 2013 checklist; recommended items to address in a clinical trial protocol and related documents.

**Additional file 2.** Educational booklet for patients with chronic obstructive pulmonary disease.

**Additional file 3.** Rehabilitation Protocol.

**Additional file 4.** Supervised session: monitoring regarding.

**Additional file 5.** Approval funding source.

## Abbreviations

COPD: Chronic obstructive pulmonary disease
PRP: Pulmonary rehabilitation programs
UFSCar: Physical Therapy Laboratory of the Federal University of São Carlos
ReBec: Brazilian Clinical Trials Registry
6MWT: Six-minute walk test
SPIRIT: Standard Protocol Items: Recommendations for Interventional Trials
mMRC: Modified Medical Research Council
kg / m2: Kilograms/meters squared
6MST: Six-minute step test
1MST: One *minute sit to stand test*
SQRQ: Saint George Respiratory Questionnaire
CAT: COPD Assessment Test
PADL: Physical activity daily living
IG: Instruction group
SG: Supervised group
SPSS: Statistical Package for the Social Sciences
FAPESP: Fundação de Amparo à Pesquisa do Estado de São Paulo
CAPES: Coordenação de Aperfeiçoamento de Pessoal de Nível Superior
