# Supplementary material for: Individualized, low-cost and accessible pulmonary rehabilitation program based on functional clinical tests for individuals with COPD—a study protocol of a randomized controlled trial
Source: Trials. 2021 May 26;22:367. doi: 10.1186/s13063-021-05267-9 (PMC8152053; doi:10.1186/s13063-021-05267-9)
Supplement: Supplementary file 2 — Additional file 2. Educational booklet for patients with chronic obstructive pulmonary disease. [file 13063_2021_5267_MOESM2_ESM.docx]

**Additional file 2.** Educational booklet for patients with chronic obstructive pulmonary disease

**
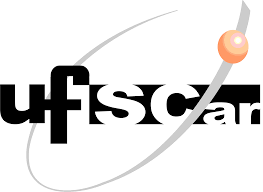
**
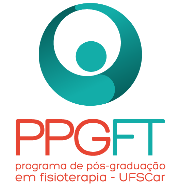
Federal University of São Carlos

Postgraduate of Physiotherapy

**EDUCATIONAL BOOKLET FOR PATIENTS WITH CHRONIC OBSTRUCTIVE PULMONARY DISEASE**

Ms. Marcela M. C. da Silva

PhD Juliano Ferreira Arcuri

PhD Valeria A. P. Di Lorenzo

****All images are prepared using the author's own source.***

**What is COPD?**

The chronic obstructive pulmonary disease (COPD) is characterized by airway (where the air passes to breathe) inflammation and airflow obstruction, Causing breathing difficulties.


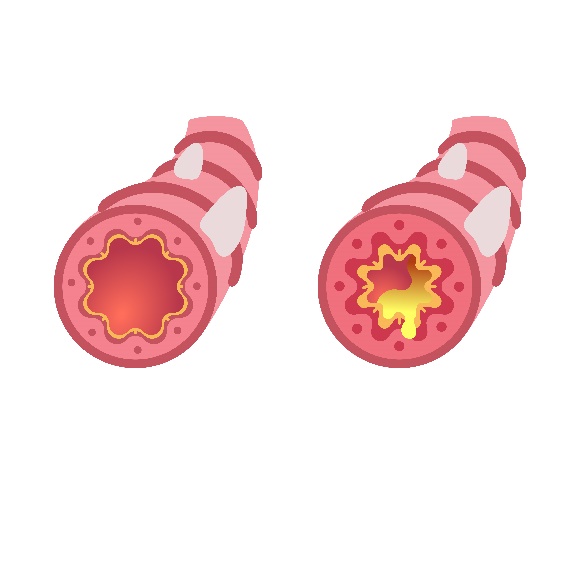


The main causes are smoking, wood or industrial stove fumes and harmful gases.


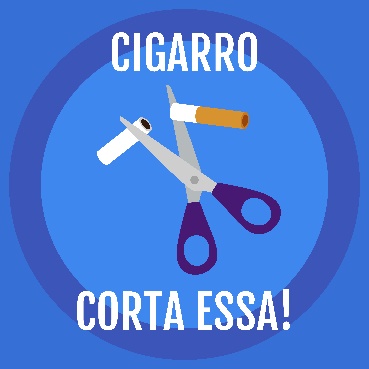


This condition causes the inflation of lung and other parts of the body such as head, upper and lower limbs.

**SOME SYMPTOMS**

- Breathlessness,

- Feeling of retained air in the lungs,

-Cough with phlegm,

- Fatigue to execute physical activities,

- Weakness in legs and arms,

- Frequent infections,

- Forgetfulness, sadness and depression,

and others.

**AND THE DIAGNOSIS?**

The diagnosis is executed by the doctor, but exposure to toxic particles or gases, associated with symptoms of chronic cough, breathlessness is commonly present. The exam that confirms the diagnosis is a spirometry "breath test".


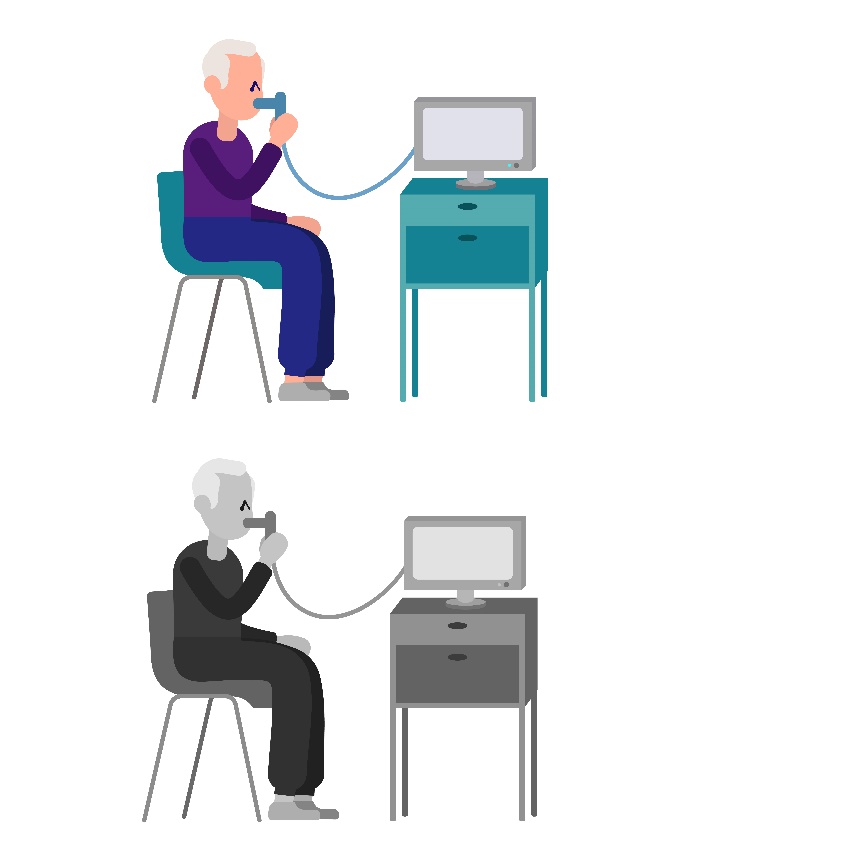


**AND THE EXACERBATION?**

The exacerbation is characterized by increase of the symptoms (breathlessness, cough with phlegm, fever, bluish nails, and others) mainly caused by infection.

***Pay attention, you need to see a doctor!**

**AND THE TREATMENT?**

Multi-professional

There is an association of care treatment that involves multi-professionals: physiotherapist, nurse, psychology, doctor, nutritionist, social assistance…

Using preventive and curative measures.

**Stop smoking**: Educational Program

Cigarettes harm your lungs. Let's stop smoking?

- Try to reduce the number of cigarettes gradually. The doctor can prescribe medication that facilitates the process. Do not forget to practice leisure and physical activities, which will greatly help!

**Vaccination (influenza and pneumococcus)**

The doctor may ask you to get some vaccines to prevent pneumonia, for example.

**Respiratory Support**

As the disease progresses, you may have insufficient oxygen in your lungs/blood. You will need supplementary oxygen.

If you have exacerbation of the disease, noninvasive ventilation (ventilation with a mask that sends air to breathe) or mechanical ventilation (breathing through a machine, with a tube that goes down to the lungs) is commonly needed.

**Medication**

The medication reduces symptoms and prevents obstruction of the airway. A different prescription may be needed depending on the individual’s health condition. So, your medication programs are prescribed especially for you! If you have any questions about your medication, ask your doctor, pharmacist or nurse for further explanations.

Oral medication such as anti-inflammatory and corticosteroids may be indicated in addition to the inhalation route, such as bronchodilators, which requires paying special attention to the correct administration technique, in addition to the intravenous via in cases of acute disease.

**ORIENTATION OF INALATION**

**Pressurized inhaler: how is it used?**

1. Remove the cap and shake vigorously;

2. Keep upright, with 4-5 cm from the mouth;

3. Start slow and deep inspiration and immediately activate the device;

4. Perform a 10-second pause;

5. Exhale normally and rinse your mouth to avoid absorption and systemic effects of the drug.

* If a new dose is prescribed, wait 15-30 sec.

**Pressurized inhaler with spacer.**

1. Remove the cap and shake vigorously;

2. Attach the aerosol to the spacer and keep it upright from the mouthpiece;

3. Adapt the mask, start slow and deep inspiration and immediately activate the device. Do 4-5 breathing cycles;

4. Take a 10 second pause on inspiration.

**Spacer hygiene**

Wash it in running water, then, in water with neutral soap and repeat again under running water. Let it dry vertically.

**Physical Activity**

COPD patients may have low physical activity level and weakness which can lead to low quality of life. When physical activity is executed daily you can improve or maintain these conditions!

**BENEFITS**

- It makes your heart stronger and healthier;
- Improves your muscle strength and weight;
- Improves your breathing;
- Reduces shortness of breath in day-to-day activities;
- Collaborates with the elimination of pulmonary secretion;
- Improves brain activity and mood;
- You will be more independent;
- Improves or maintains bone density;
- Strengthens the immune system.
- Benefits of physical activity.

**CARE TO PERFORM PHYSICAL ACTIVITY**

- Administrate the correct medication prescribed by the doctor in times of greater dyspnea;
- Stop if you have a fever or infection or any discomfort such as chest pain and dizziness and look for a professional;
- Frequency: 3 to 5 times a week;
- Duration: from 30 to 50 minutes;
- Intensity: For each exercise guided below, the physiotherapist will teach you what intensity you should maintain to be safe. Remember to achieve from the most moderate to intense fatigue (Borg's Dyspnea Scale).

MODIFIED BORG SCALE- Krendrick et al.

| 0 | No breathlessness at all |
| --- | --- |
| 0,5 | Very, very slight |
| 1 | very slight |
| 2 | Slight breathlessness |
| 3 | Moderate |
| **4** | **Somewhat severe** |
| **5** | **Severe breathlessness** |
| 6 |  |
| 7 | Very severe breathlessness |
| 8 |  |
| 9 | Very, very severe |
| 10 | Maximal |

**You must maintain a 4-5 fatigue.**

**HOME EXERCISE PROGRAM**

**START: / /**

**THE END: / /**

The program is divided into various steps.

Remember that you can take breaks between each step.

**STEP 1:** Consists of warm up exercise: slow exercise such as walking for 3 to 5 minutes.


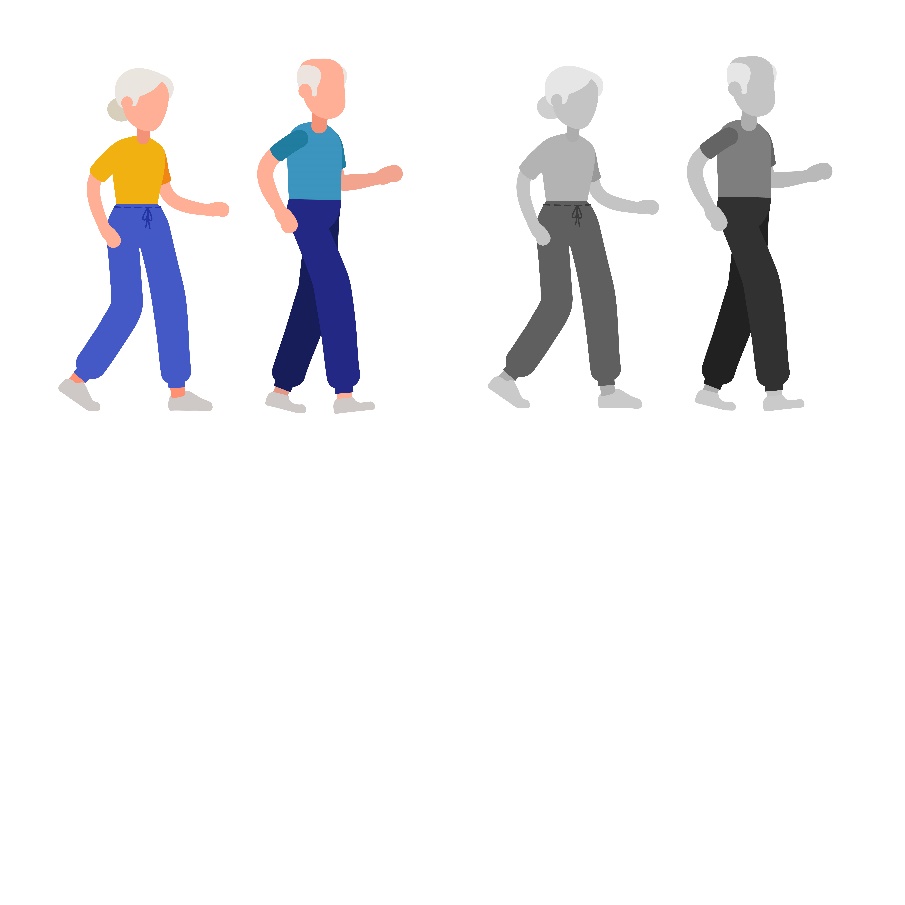


**SLOW**

**Household report: _______________________________________________________________________________________________________________________________________________________________________________________________________________________________________________**

**BREAK: _____________________________________________________**

**STEP 2: Consists of aerobic exercise.**

**WALKING:** Performed on flat ground initially


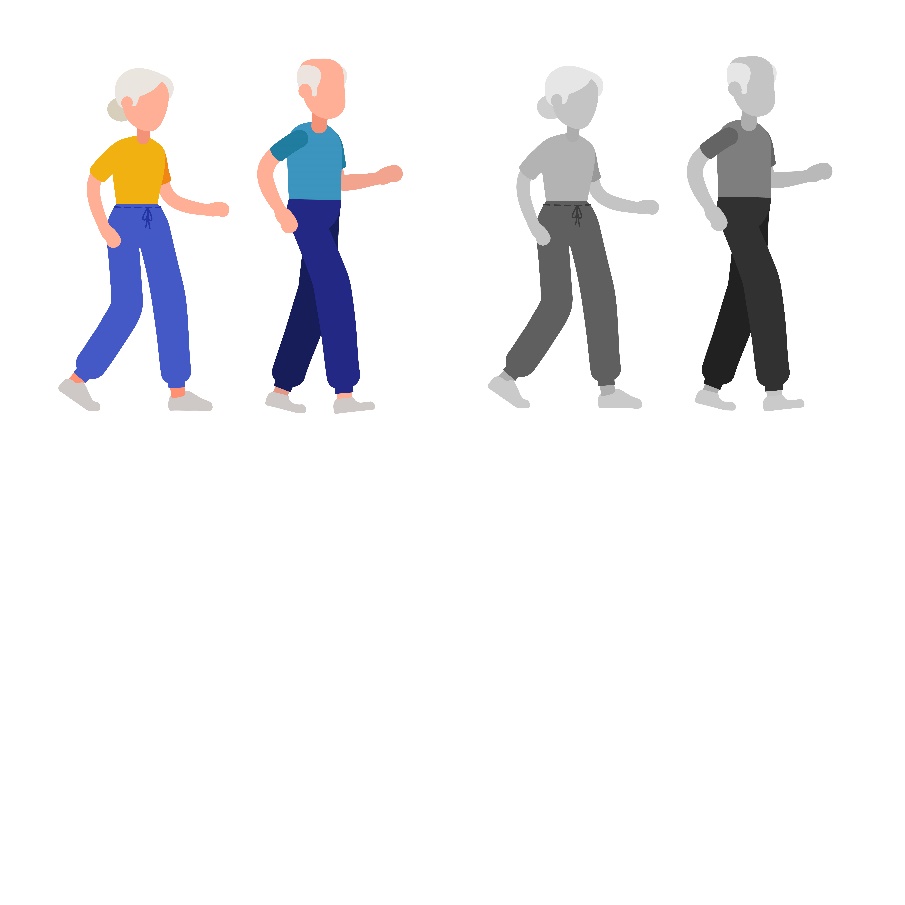


| Prescription | | | | | | | | |
| --- | --- | --- | --- | --- | --- | --- | --- | --- |
| WEEKs | 1 | 2 | 3 | 4 | 5 | 6 | 7 | 8 |
| Time |  |  |  |  |  |  |  |  |
| Distance walked |  |  |  |  |  |  |  |  |

Household report

WEEK 1:___________________________________________

WEEK 2:___________________________________________

WEEK 3:___________________________________________

WEEK 4___________________________________________

WEEK 5:____________________________________________

WEEK 6:____________________________________________

WEEK 7:____________________________________________

WEEK 8:____________________________________________

**BREAK: _____________________________________________________**

**STEP UP AND DOWN**


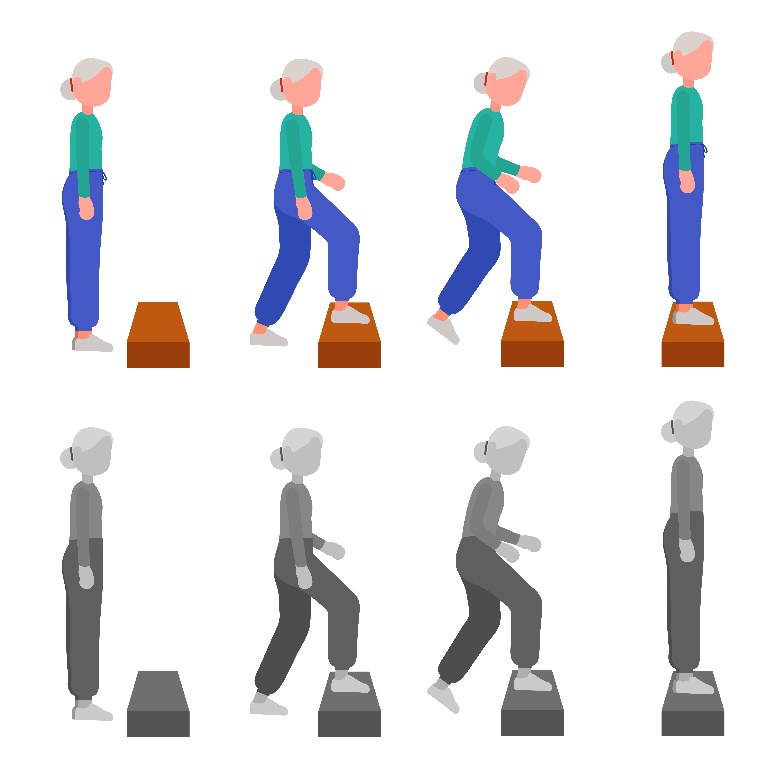


| Prescription | | | | | | | | |
| --- | --- | --- | --- | --- | --- | --- | --- | --- |
| WEEKs | 1 | 2 | 3 | 4 | 5 | 6 | 7 | 8 |
| Time |  |  |  |  |  |  |  |  |
| Minimum number of repetitions |  |  |  |  |  |  |  |  |

Household report

WEEK 1:____________________________________________

WEEK 2:____________________________________________

WEEK 3:____________________________________________

WEEK 4_____________________________________________

WEEK 5:____________________________________________

WEEK 6:____________________________________________

WEEK 7:____________________________________________

WEEK 8:____________________________________________

**BREAK: ____________________________________________________**

**SIT TO STAND MOVEMENTS**


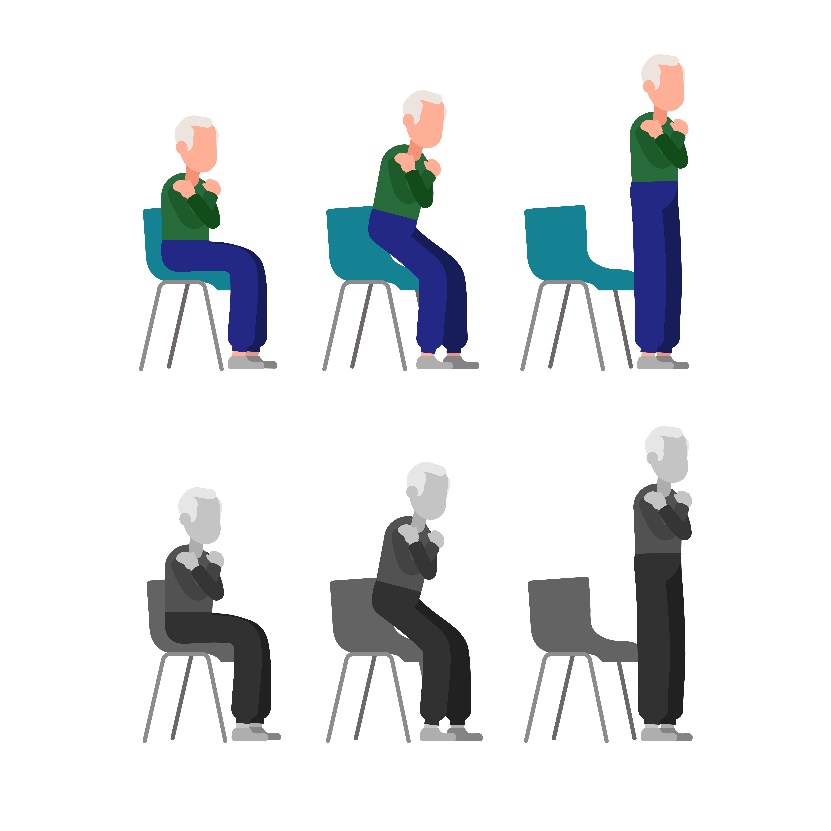


| Prescription | | | | | | | | |
| --- | --- | --- | --- | --- | --- | --- | --- | --- |
| WEEKs | 1 | 2 | 3 | 4 | 5 | 6 | 7 | 8 |
| Time |  |  |  |  |  |  |  |  |
| Minimum number of repetitions |  |  |  |  |  |  |  |  |

Household report

WEEK 1:____________________________________________

WEEK 2:____________________________________________

WEEK 3:____________________________________________

WEEK 4___________________________________________

WEEK 5:____________________________________________

WEEK 6:____________________________________________

WEEK 7:____________________________________________

WEEK 8:____________________________________________

**BREAK: ____________________________________________________**

**STEP 3: Upper limbs strengthening.**

**A**. Use food packages with equivalent weight: execute standing or sitting, performed in diagonal movements.


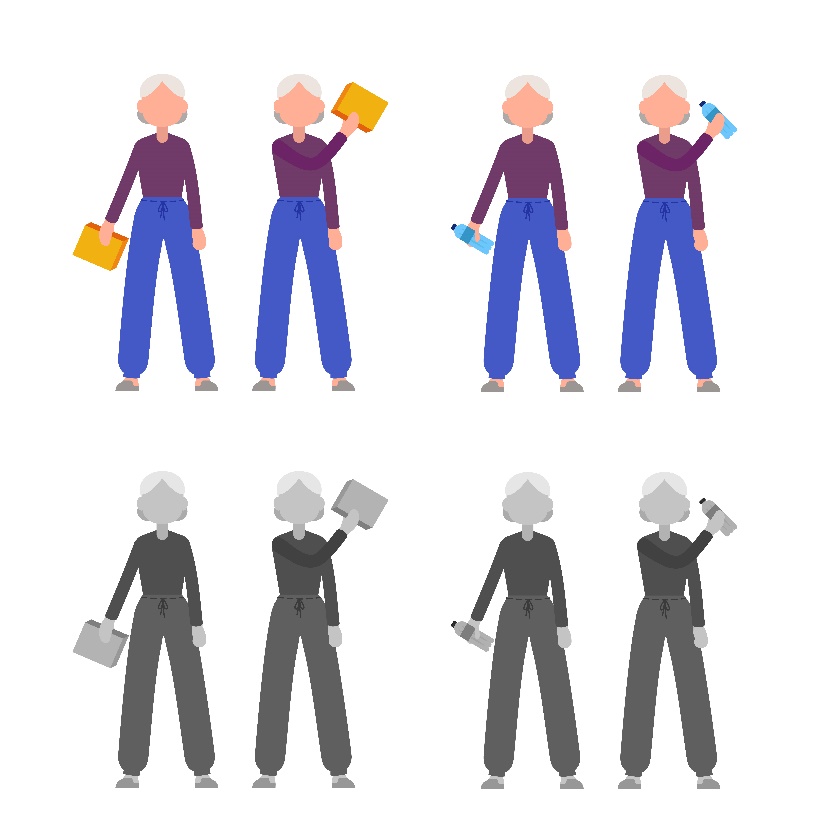


| Prescription | | | | | | | | |
| --- | --- | --- | --- | --- | --- | --- | --- | --- |
| WEEKs | 1 | 2 | 3 | 4 | 5 | 6 | 7 | 8 |
| Weight |  |  |  |  |  |  |  |  |
| Repetition |  |  |  |  |  |  |  |  |
| Series |  |  |  |  |  |  |  |  |

Household report

WEEK 1:____________________________________________

WEEK 2:____________________________________________

WEEK 3:____________________________________________

WEEK 4:____________________________________________

WEEK 5:____________________________________________

WEEK 6:____________________________________________

WEEK 7:____________________________________________

WEEK 8:____________________________________________

**BREAK: _____________________________________________________**

**Complementary exercise: push-ups against the wall**

Don’t forget to associate de respiratory exercise during the flexion (expiration) and extension (inspiration).


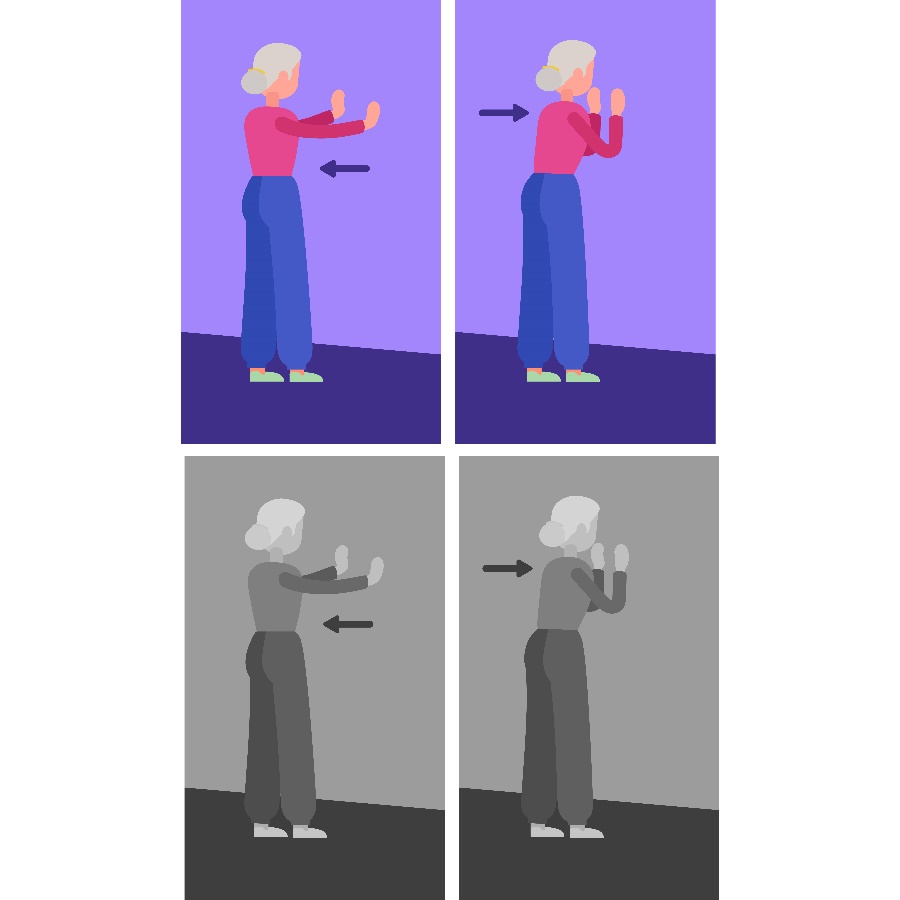


| Prescription | | | | | | | | |
| --- | --- | --- | --- | --- | --- | --- | --- | --- |
| WEEKs | 1 | 2 | 3 | 4 | 5 | 6 | 7 | 8 |
| Repetition |  |  |  |  |  |  |  |  |
| Series |  |  |  |  |  |  |  |  |

Household report

WEEK 1:____________________________________________

WEEK 2:____________________________________________

WEEK 3:____________________________________________

WEEK 4_____________________________________________

WEEK 5:____________________________________________

WEEK 6:____________________________________________

WEEK 7:____________________________________________

WEEK 8:____________________________________________

**STEP 4: Stretching**

Remain in each position for 20 seconds.

In the sitting position, stretch the cervical (“lateral neck movement”) upper limb ("arm to the side and arm back") and lower limbs (standing or lying position) ("hold the knees" and "hold the feet").


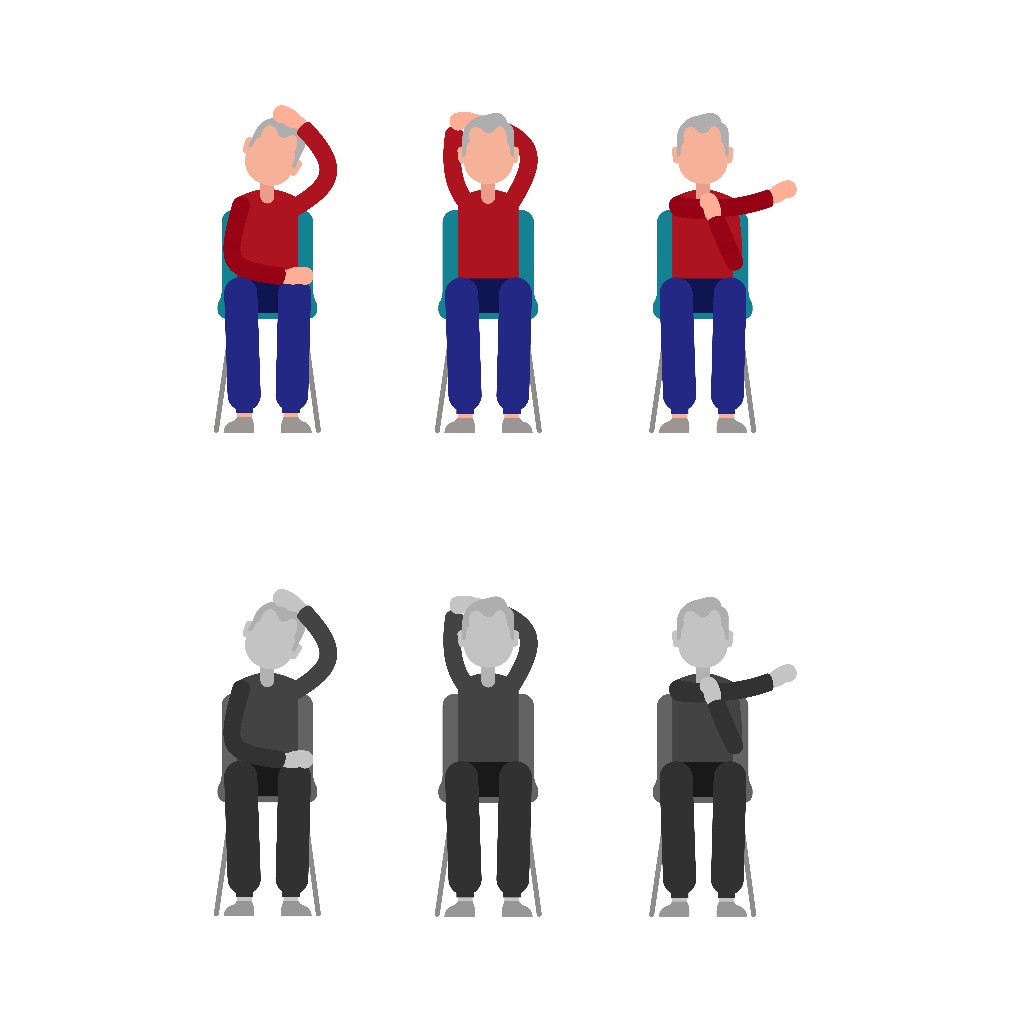


Neck

Upper limbs

Lower limbs


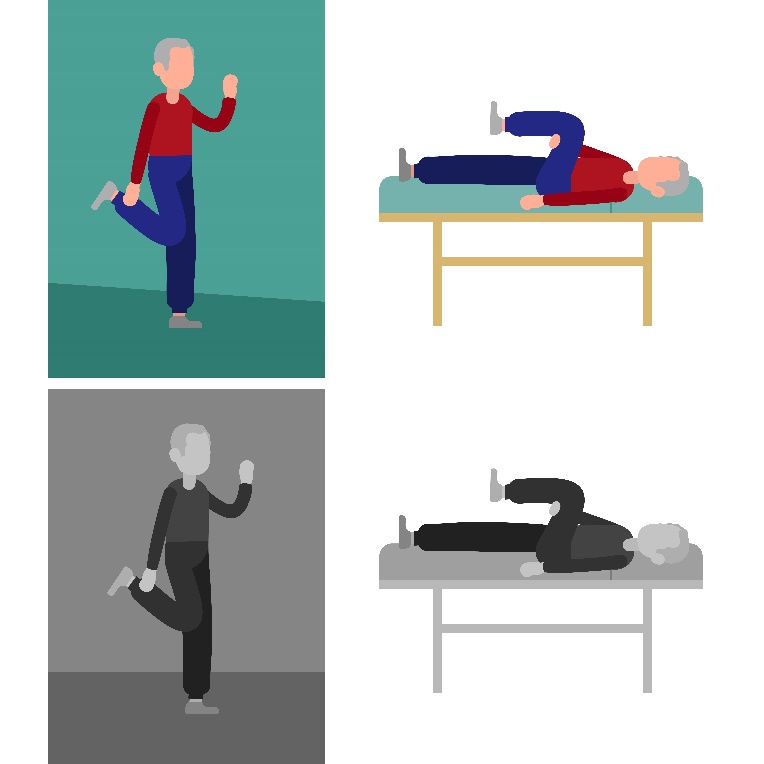


**STEP 5: Relation**

For 5 to 10 minutes rest in bed listening to relaxing music and controlled respiration.

**Lung hygiene techniques**

Mucociliary activity (pulmonary cilia that help clear the secretion) can be affected by smoking, use of oxygen, and dehydration (always drink water properly!). If there is also a lung infection condition, the airway may become inflamed and produce more secretion.

Excessive secretion can increase cough and fatigue and shortness of breath. If the secretion is not removed from the lung, it can cause further inflammation and damage to your lungs.

There are techniques that can help to remove this secretion from the lung, this subject should be discussed, guided or performed by a respiratory physiotherapist.

**Huffing**

Through forced exhalation with the open glottis (exhaling as if “puffing glasses”), the secretion is removed.

**Cough**

Coughing is the effective way to remove secretion, without making maximum efforts.

Take a calm breath, then immediately execute a forced expiration. You will feel that the abdominal muscles will expel the secretion.

**Doubts?**

**Contact your physiotherapist!**
