# Supplementary material for: Individualized, low-cost and accessible pulmonary rehabilitation program based on functional clinical tests for individuals with COPD—a study protocol of a randomized controlled trial
Source: Trials. 2021 May 26;22:367. doi: 10.1186/s13063-021-05267-9 (PMC8152053; doi:10.1186/s13063-021-05267-9)
Supplement: Supplementary file 3 — Additional file 3. Rehabilitation Protocol. [file 13063_2021_5267_MOESM3_ESM.docx]

**Additional file 3.** Rehabilitation Protocol

| **Rehabilitation protocol**  **Patient name:** | | | | | | | | |
| --- | --- | --- | --- | --- | --- | --- | --- | --- |
| **step height:** | | | **Walking place:** | | | **Strengthening sitting or standing:** | | |
|  | | | | | | | | |
| STEPS | **WEEK 1**  Data: | **WEEK 2**  Data: | **WEEK 3**  Data: | **WEEK 4**  Data: | **WEEK 5**  Data: | **WEEK 6**  Data: | **WEEK 7**  Data: | **WEEK 8**  Data: |
| **Warm up exercise** | 3-5 minutes: light exercise | 3-5 minutes: light exercise | 3-5 minutes: light exercise | 3-5 minutes: light exercise | 3-5 minutes: light exercise | 3-5 minutes: light exercise | 3-5 minutes: light exercise | 3-5 minutes: light exercise |
| Prescription |  |  |  |  |  |  |  |  |
| INTERVAL ____minutes | | | | | | | | |
| AEROBIC EXERCISE: Keep the BORG scale 3-5 points | | | | | | | | |
| Walk | 5 minutes 80% | 8 minutes 80% | 10 minutes 90% | 12 minutes 100% | 13 minutes 100% | 15 minutes 100% | 15 minutes 100% | 15 minutes 100% |
| Prescription |  |  |  |  |  |  |  |  |
| INTERVAL | | | | | | | | |
| Up and down step | 5 minutes 80% | 6 minutes 80% | 7 minutes 90% | 8 minutes 100% | 9 minutes 100% | 10 minutes 100% | 11 minutes 100% | 12 minutes 100% |
| Prescription |  |  |  |  |  |  |  |  |
| INTERVAL ____minutes | | | | | | | | |
| Sit to stand | 1 minutes 80% | 2 minutes 80% | 2 minutes 90% | 3 minutes 90% | 3 minutes 100% | 4 minutes 100% | 5 minutes 100% | 5 minutes 100% |
| Prescription |  |  |  |  |  |  |  |  |
| INTERVAL ____minutes | | | | | | | | |
| Upper limbs strengthening | 50%10RM | 50%10RM | 75%10RM | 75%10RM | 75%10RM | 100%10RM | 100%10RM | 100%10RM |
| Prescription |  |  |  |  |  |  |  |  |
| INTERVAL ____minutes | | | | | | | | |
| Flexion against the wall |  |  |  |  |  |  |  |  |
| Prescription |  |  |  |  |  |  |  |  |
| Stretching |  |  |  |  |  |  |  |  |
| Relaxation |  |  |  |  |  |  |  |  |
