# Supplementary material for: Individualized, low-cost and accessible pulmonary rehabilitation program based on functional clinical tests for individuals with COPD—a study protocol of a randomized controlled trial
Source: Trials. 2021 May 26;22:367. doi: 10.1186/s13063-021-05267-9 (PMC8152053; doi:10.1186/s13063-021-05267-9)
Supplement: Supplementary file 4 — Additional file 4. Supervised session: monitoring regarding. [file 13063_2021_5267_MOESM4_ESM.docx]

**Additional file 4.** Supervised session: monitoring

| **WEEK** | **PRE** | | | | **POST WALKING** | | | **POST STEP** | | | **POST SIT TO STAND** | | | **POST STRENGTHENING** | | | **POST** | | | |
| --- | --- | --- | --- | --- | --- | --- | --- | --- | --- | --- | --- | --- | --- | --- | --- | --- | --- | --- | --- | --- |
|  | RR | SpO2 | BORG | Arterial pressure | RR | SpO2 | BORG | RR | SpO2 | BORG | RR | SpO2 | BORG | RR | SpO2 | BORG | RR | Arterial pressure | SpO2 | BORG |
| **1** |  |  |  |  |  |  |  |  |  |  |  |  |  |  |  |  |  |  |  |  |
| **2** |  |  |  |  |  |  |  |  |  |  |  |  |  |  |  |  |  |  |  |  |
| **3** |  |  |  |  |  |  |  |  |  |  |  |  |  |  |  |  |  |  |  |  |
| **4** |  |  |  |  |  |  |  |  |  |  |  |  |  |  |  |  |  |  |  |  |
| **5** |  |  |  |  |  |  |  |  |  |  |  |  |  |  |  |  |  |  |  |  |
| **6** |  |  |  |  |  |  |  |  |  |  |  |  |  |  |  |  |  |  |  |  |
| **7** |  |  |  |  |  |  |  |  |  |  |  |  |  |  |  |  |  |  |  |  |
| **8** |  |  |  |  |  |  |  |  |  |  |  |  |  |  |  |  |  |  |  |  |
