# Supplementary figures and images for: Individualized, low-cost and accessible pulmonary rehabilitation program based on functional clinical tests for individuals with COPD—a study protocol of a randomized controlled trial
Source: Trials. 2021 May 26;22:367. doi: 10.1186/s13063-021-05267-9 (PMC8152053; doi:10.1186/s13063-021-05267-9)

**Additional file 5: Aproval financial support**


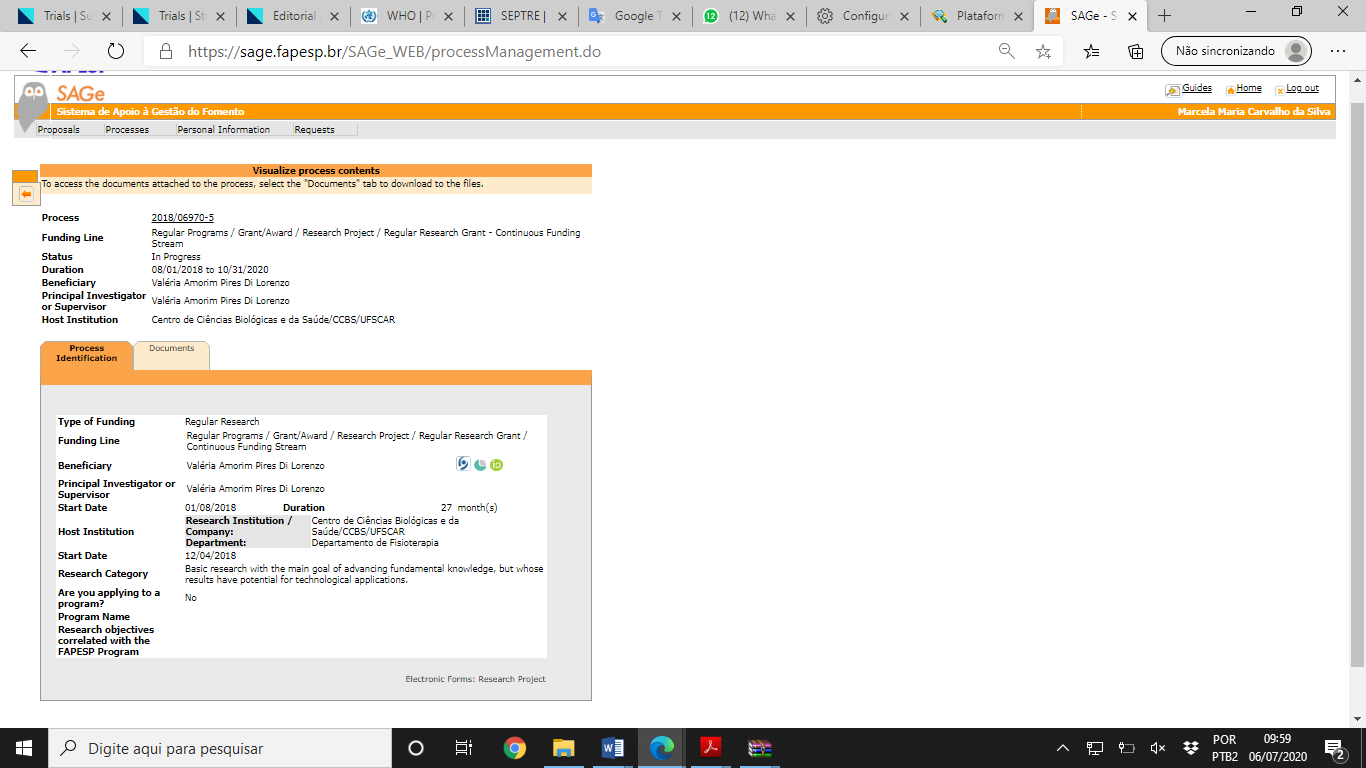

Supplement: Supplementary file 5 — Additional file 5. Approval funding source. [file 13063_2021_5267_MOESM5_ESM.docx]
